# Genomic and AntiSMASH Analyses of Marine-Sponge-Derived Strain *Aspergillus niger* L14 Unveiling Its Vast Potential of Secondary Metabolites Biosynthesis

**DOI:** 10.3390/jof8060591

**Published:** 2022-05-31

**Authors:** Ping Wang, Shuang Xu, Yuqi Tang, Hong Wang, Xuelian Bai, Huawei Zhang

**Affiliations:** 1 School of Pharmaceutical Sciences, Zhejiang University of Technology, Hangzhou 310014, China; wangping_1009@163.com (P.W.); 17853509869@163.com (S.X.); t17670659912@163.com (Y.T.); hongw@zjut.edu.cn (H.W.); 2 College of Life and Environmental Sciences, Hangzhou Normal University, Hangzhou 311121, China; baixl2012@163.com

**Keywords:** *Aspergillus niger*, symbiotic microorganism, genome sequencing, antiSMASH, natural product, biosynthetic potential

## Abstract

*Aspergillus niger* is one of the most important sources of secondary metabolites (SMs), with a wide array of pharmacological effects, including anti-inflammatory, antitumor, immunomodulatory and antioxidant effects. However, the biosynthetic analysis of these bioactive components has been rarely reported owing to the lack of high-quality genome sequences and comprehensive analysis. In this study, the whole genome of one marine-sponge-derived strain *A. niger* L14 was sequenced and assembled as well as in-depth bioinformatic analysis. The results indicated that the sequence assembly of strain L14 generated one high-quality genome with a total size of 36.1 Mb, a G + C content of 45.3% and an N50 scaffold of 4.2 Mb. Gene annotation was extensively deployed using various BLAST databases, including non-redudant (Nr) protein sequence, nucleotide (Nt) sequence, Swiss-Prot, Gene ontology (GO), Kyoto Encyclopedia of Genes and Genomes (KEGG) and Clusters of Orthologous Groups (COG) as well as Pathogen Host Interactions (PHI) and Carbohydrate-active enzymes (CAZy) databases. AntiSMASH analysis revealed that this marine strain harbors a total of 69 SMs biosynthesis gene clusters (BGCs), including 17 PKSs, 18 NRPSs, 21 NRPS-likes, 9 terpenes, 2 indoles, 1 betalactone and 1 siderophore, suggesting its biosynthetic potential to produce a wide variety of SMs. These findings will assist in future investigations on the genetic basis of strain L14 and provide insights into its new bioactive SMs for new drug discovery.

## 1. Introduction

Marine-derived *Aspergillus* is appraised as a favorable source for discovering new bioactive secondary metabolites (SMs). Currently, marine-derived *Aspergillus* has been reported about two hundred SMs, which is one of the most widely studied marine fungi in the genus [1]. It mainly includes structural types, such as flavonoids, alkaloids, phenylpropanoids, terpenes, quinones, cyclic peptides and other compounds. Some of these metabolites have shown anti-inflammatory, anti-tumor, antibacterial, free radical scavenging and other biological activities, which may be promising resources for new therapeutic drugs [2,3,4]. The classical pre-genomics assumed that an individual microbial strain could produce only a limited number of SMs, but the first genome sequences *Aspergillus* published in the 2000s [5] and the subsequent explosion of genome sequencing data in the 2010s demonstrated that these microorganisms have the genetic capability to produce significant numbers of compounds. These unknown metabolic pathways are likely to encode numerous bioactive molecules. One of the most commonly used tools, antiSMASH (antibiotics and Secondary Metabolites Analysis Shell) has had a major impact on genome-to-genome correlation, query and prediction of natural product synthetic gene clusters [6,7].

It is well known that *Aspergillus niger* is an excellent producer of valuable metabolites, including organic acids (e.g., citric acid, itaconic acid and tensyuic acid) and various enzymes (e.g., polysaccharide-degrading enzymes, amylolytic enzymes) [8,9,10,11]. In our continuous search of bioactive SMs from marine fungi, one fungal strain L14 was obtained from marine sponge *Reniera japonica* and its chemical study led to isolation of 14 known SMs (fonsecinone A, isoaurasperone A, aurasperone E, asperpyrone C, asperpyrone D, aurasperone G, nigerone, asperpyrone B, tensyuic acid E, tensyuic acid F, 9-hydroxyhexylitaconic acid-1-methyl ester, asperlone B, pyrophen and 2-benzyl-γ-pyrone), most of which are polyketide derivatives (Figure S1). However, these findings were not exciting, since other type SMs were not obtained. It might be ascribed to that fact that most of SM biosynthetic gene clusters (BGCs) in strain L14 were silent or expressed at low levels under conventional culture conditions. In order to better understand and exploit the biosynthetic potential of SMs in depth for new drug discovery, whole-genome sequencing and bioinformative analysis with antiSMASH annotation of strain L14 were extensively carried out in this work.

## 2. Materials and Methods

### 2.1. Strain Source

Strain L14 was isolated from one specimen (MNP-2016) of *R. japonica*, which was collected in September 2016 from the Xinghaiwan Coast of Dalian City, Liaoning Province, China [12]. One suspension of culture containing its mycelia in potato dextrose broth (PDB) supplemented with glycerol (20% *v*/*v*) was stored at −80 °C.

### 2.2. Phylogenetic Analysis

For phylogenetic analysis, strain L14 was cultivated in PDB medium at 28 °C for 3 days followed by 18*S* rRNA gene amplicon sequencing. Its 18*S* rRNA sequence with 1680 bp had been submitted to GenBank in NCBI and an accession number MF093522 was acquired. Forty strains with high similarity in NCBI database were selected to construct the phylogenetic tree using MEGA 7.0.26 (Table S1), and the phylogenetic tree was delineated via a neighbor-joining mode which was constructed using the Tamura3 parameter model with 1000 bootstrap replicates.

### 2.3. DNA Extraction and Whole Genome Sequencing

Genomic DNA of strain L14 was extracted with Gentra Puregene Yeast/Bact. Kit (Qiagen, Valencia, CA, USA) officially recommended by Nanopore [13]. The next-generation and third-generation sequencing technologies were, respectively, utilized Illumina HiSeq 2500 platform and PacBio Sequel platform. DNA libraries of a certain concentration and volume were added to each individual flow cell and the flow cells were transferred to a Grid ION X5 sequencer (Nanopore, Oxford, UK) for real-time single-molecule sequencing. Through a variety of methods, including Homology, SNAP, Augustus, coding genes, repetitive sequences, non-coding RNA, etc., were predicted to obtain the composition of the sequenced strain genome. Repeat sequence prediction was performed by Proteinmask, repeatmasker and trf. The tRNA region was predicted by tRNAscan software, rRNA was predicted by RNAmmer software, miRNA and snRNA were achieved by comparison with Rfam database.

### 2.4. Genome Assembly

Unicycler software (https://github.com/rrwick/Unicycler, accessed on 10 September 2020) was used for assembly, high-quality data and debug multiple assembly versions were used to select the optimal assembly results [14]. After sequencing, the quality control of data included raw data and clean data quality control files. Clean data were high-quality data obtained by removing connectors and filtering low-quality data according to the original off-line data. Contaminated or low-quality reads, as well as the proportion of N at one read greater than 5% were removed.

### 2.5. Gene Annotation

Functional genes in whole genome of strain L14 were, respectively, annotated by Pathway, Nr (NCBI non-redundant protein sequences), COG (Cluster of Orthologous Groups of proteins) and KEGG (Kyoto Encyclopedia of Genes and Genomes), as well as GO (Gene Ontology). Through BLAST, the protein sequence was aligned to protein database Nr, GO, KEGG and COG to obtain the highest similarity with a given unigene of the protein containing the function annotation information [15]. Based on Nr annotation information, GO annotation information was obtained by using Blast2GO software. GO functional classification statistics were performed by using WEGO software [16] and KEGG was used to investigate the complex behavior of genes in biology [17]. According to the KEGG annotation information, the pathway annotation of all unigenes was obtained. COG is a database for orthologous classification of gene products. Each COG protein was assumed to be derived from an ancestor protein [18]. The COG database was constructed based on the coding proteins and phylogenetic relationships of bacteria, algae and eukaryotes with complete genomes. The protein sequence was compared by the COG database, the possible function of the protein was also predicted.

### 2.6. Additional Annotation

Diamond software was used to align the amino acid sequences of the target species with the CAZy database (Carbohydrate-Active Enzymes Database) [19]. Annotation results were screened to ensure biological significance. Meanwhile, each gene in the database contains nucleic acid and amino acid sequences, as well as a detailed description of the protein function predicted during host infection. BLAST software was used to compare the amino acid sequence of the target species with the PHI (Pathogen Host Interactions Database) database (E-value < 0.00001), annotation results were achieved by combining the gene of the target species with its corresponding function annotation information. Our selection criteria for labeling were retaining the comparison results with the highest scores and the query coverage rate was greater than 80%.

### 2.7. Secondary Metabolic Gene Cluster Analysis

Based on gbk files, nucleic acid sequence and protein sequence, antiSAMSH 6.0 software was used to find secondary metabolic clusters [20]. NCBI database searched for genomic information of strain L14. Generally, genes involved in biosynthetic enzymes in secondary metabolic pathways are clustered on chromosomes. Secondary metabolic gene clusters were classified into 24 categories. The most common secondary metabolic gene clusters are type I, II and III polyketides synthase (PKS) [21,22] and non-ribosomal peptides synthase (NRPS) [23].

## 3. Results and Discussion

### 3.1. Morphology and Classification of Strain L14

**Figure 1:**
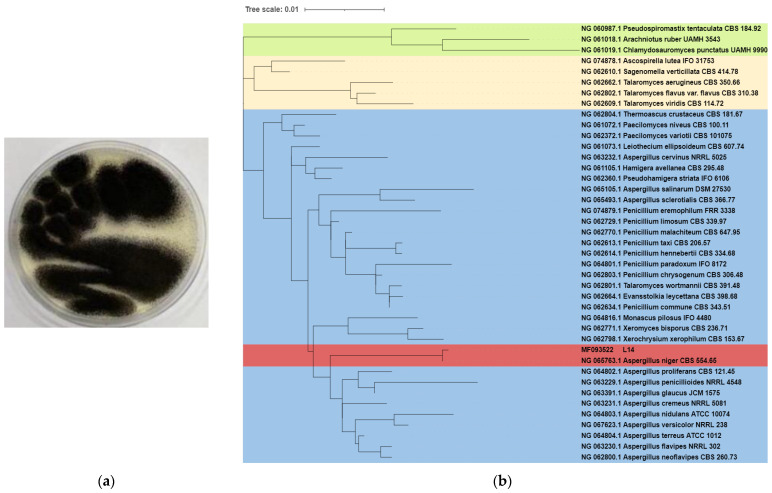
Colony morphology (**a**) and 18*S* rRNA-based phylogenetic tree (**b**) of strain L14.

The white mycelia of strain L14 grew from a potato dextrose agar (PDA) medium after incubation for 3 d at 28 °C, and gradually became darker and its black spores were produced on the seventh day (Figure 1a). Phylogenetic analysis displayed strain L14 had 100% of the 18S rRNA sequence of the reference strain A. niger CBS 554.65 (Figure 1b), suggesting that this isolate unambiguously belongs to the species of A. niger (Table S1).

### 3.2. Genomic Assembly and Analysis

**Figure 2:**
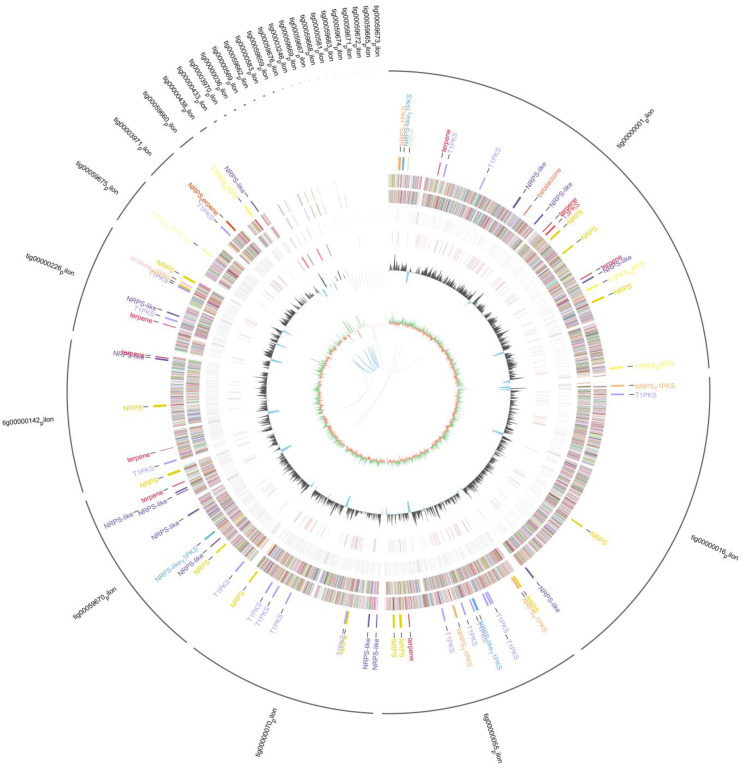
Genome diagram of strain L14 (from inside to outside: the first circle of blue lines shows in-paralog pairs better hits between each other, the E-value between pairs is lower than 1 × 10^−5^; the second circle shows GC skew, green is the positive part of GC skew, orange is the negative part of GC skew; the third circle shows the GC content; the fourth circle shows ncRNA; the fifth circle shows the repeat; the sixth circle and the seventh circle show the CDS annotation information, and different colors represent different COG annotation classifications; the sixth circle is the CDS in the negative chain; the seventh circle is the CDS in the positive chain; the eighth circle shows the SMs, where the corresponding gene cluster names are marked; the outermost circles show the various scaffolds).

The genome sequencing of strain L14 yielded a sequence with a length of 36,104,262 bp with a G + C content of 49.3% and N50 of 4,203,503 bp. After base calling and Bcl2Fastq transforming, 42,699,652 clean reads were achieved. By extensive search, the genome size of *A. niger* strains deposited in NCBI database ranged from 34 Mb to 36 Mb; the GC content is approximate 50% (Table 1). As shown in Figure 2, the genome diagram of strain L14 consists of nine circles. As such, 11,524 coding genes were predicted, accounting for 43.23% of the genome. Protein mask, REPEAT masker and TRF were used to predict repeat size (167,544 bp). Repeat masker showed the repeat size is 474,020 bp, 1.31% in Genome. TRF showed the repeat size is 224,849 bp, 0.62% in Genome. The number of long terminal repeat was 166, occupying 0.21%. The number of long interspersed elements was 89, occupying 0.14%. The number of DNA transposons was 82, occupying 0.09%; there were non-coding RNAs, 80 rRNAs, 34 tRNAs and 296 tRNAs (Table 2).

### 3.3. Functional Gene Annotation

**Figure 3:**
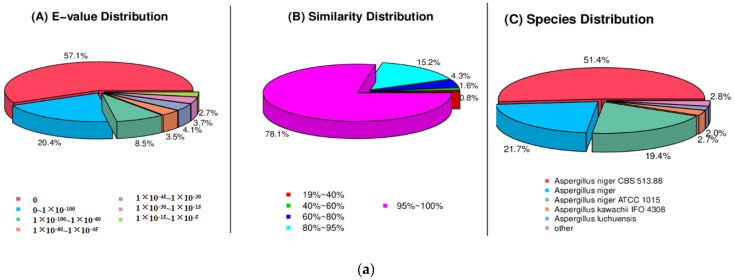

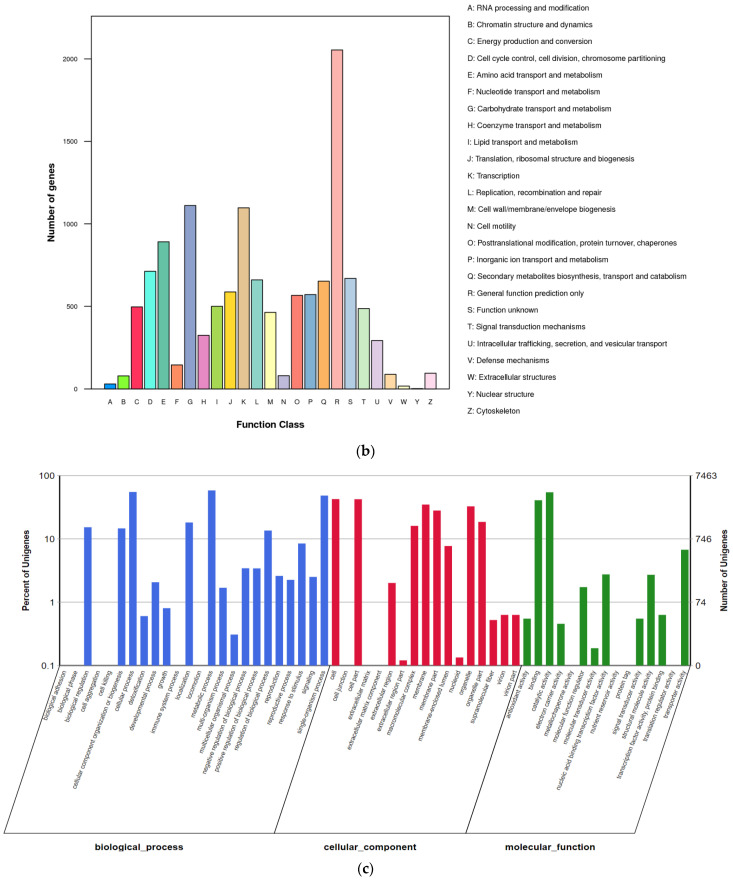

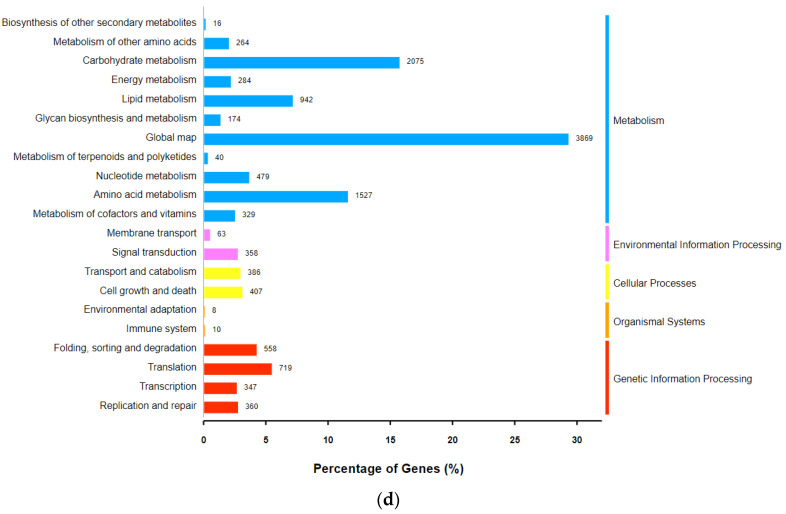
E-value, similarity and species distribution of strain L14 annotated by Nr database; (**a**), cluster of orthologous groups of proteins (COG) (**b**), gene ontology (GO) (**c**) and Kyoto encyclopedia of genes and genomes (KEGG) (**d**).

The largest number of functional genes in strain L14 was determined as 11,300 genes/98.06% using the Nr database followed by Nt (11,228 genes/97.43%), Swiss-PROt (8147 genes/70.70%), KEGG (7818 genes/67.84%), COG (6162 genes/53.47%) and GO (7464 genes/64.77%). Among these 11,300 genes, 8829 genes have more than 95% similarity with the gene sequences in the Nr database. The results of species annotation showed that the sequence was more likely to be of *A. niger* strains CBS 523.88 (Figure 3a). It is noteworthy that strain L14 contains 25 Ortholog clusters with various functions, including defense mechanisms, energy production and conversion (Figure 3b). COG analysis suggests that the largest number of genes (2054) belongs to the family of “general function prediction only” followed by “carbohydrate transport and metabolism” (1111), “transcription” (1097) and “amino acid transport and metabolism” (891). Based on GO assignment, 7464 genes were categorized into 90 functional groups. In terms of biological process, genes were detected to be involved in metabolic processes (4339), cellular processes (4103) and single-organism processes (3596). Meanwhile, the number of genes involved in cellular components were cell (3166), cell part (3151), membrane (2594) and organelle (2432). The molecular function revealed that 4056 genes were involved in catalytic activity (4065), followed by binding (3035) (Figure 3c). KEGG annotation obtained global map (3869), carbohydrate metabolism (2075) and amino acid metabolism (1527), which contained a relatively large number of genes (Figure 3d). These findings suggested the presence of an enriched and varied array of carbohydrates and amino metabolism functions that enable higher energy conversion efficiency. Numerous global map function genes indicated that strain L14 can be a very considerable research object for the study of global regulation.

### 3.4. Additional Annotation

**Figure 4:**
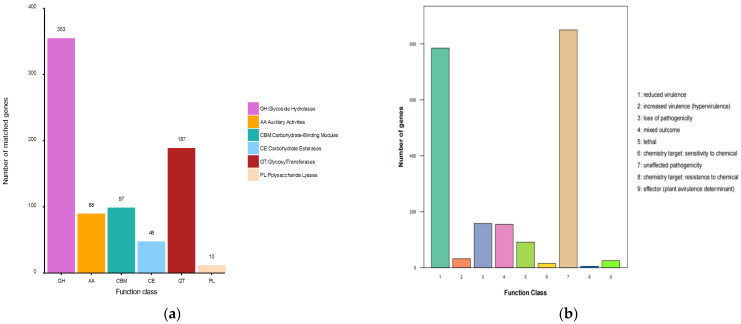
CAZy (**a**) and PHI (**b**) profiles of strain L14.

Six carbohydrate-active enzyme (CAZyme) families were identified from the whole genome of strain L14 and consisted of glycol side hydrolases (GH) with 353 genes, glycosyl transferase (GT) with 187 genes, carbohydrate-binding module (CBMs) with 97 genes, carbohydrate esterase (CE) with 46 genes and polysaccharide lyase (PL) with 10 genes, as well as auxiliary activity (AA) with 88 genes (Figure 4a). Particularly, this marine strain possesses the largest number of GHs, suggesting its robust ability to break down lignocellulose [24]. The pathogen–host interaction (PHI) analysis of strain L14 resulted in identification of 2116 genes classified into nine groups, in which the largest is “unaffected pathogenicity” with 850 genes, followed by “reduced virulence” (785 genes), “loss of pathogenicity” (158 genes), “mixed outcome” (155 genes), “lethal” (91 genes), “increased virulence (hypervirulence)” (32 genes), “effector (plant avirulence determinant)” (25 genes), “chemistry target: sensitivity to chemical” (15 genes) and “chemistry target: resistance to chemical” (5 genes) (Figure 4b). The detailed information for these genes is comprehensively summarized in Table S2.

### 3.5. AntiSMASH Analysis

**Figure 5:**
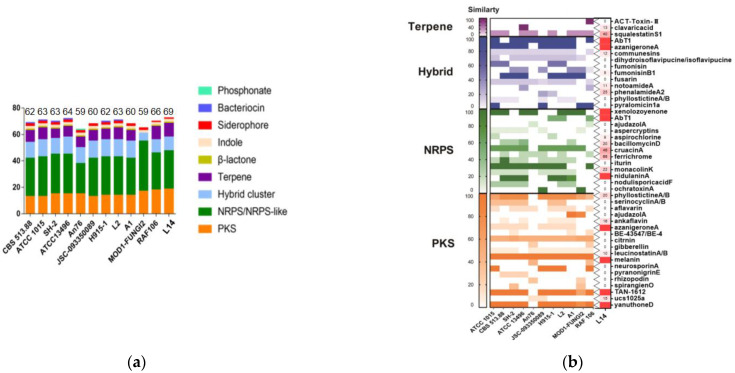
The number and types of secondary metabolite BGCs (**a**) and their putative products (**b**) in strain L14 and other *A. niger* strains deposited in antiSMASH database.

**Figure 6:**
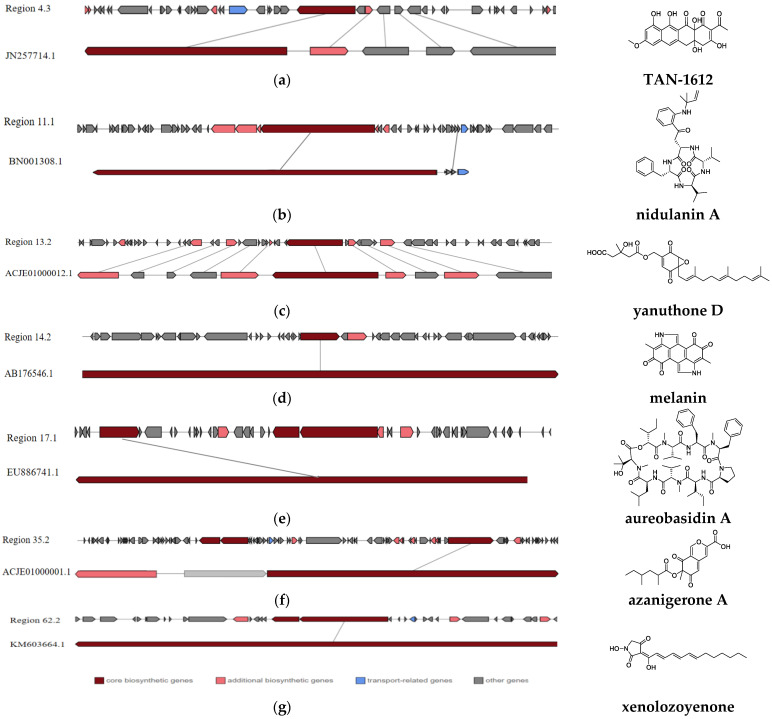
Seven identified BGCs with over 93% identity in strain L14 responsible for biosynthesis of TAN-1612 (**a**), nidulanin A (**b**), yanuthone D (**c**), melanin (**d**), aureobasidin A (**e**); azanigerone A (**f**) and xenolozoyenone (**g**).

**Figure 7:**
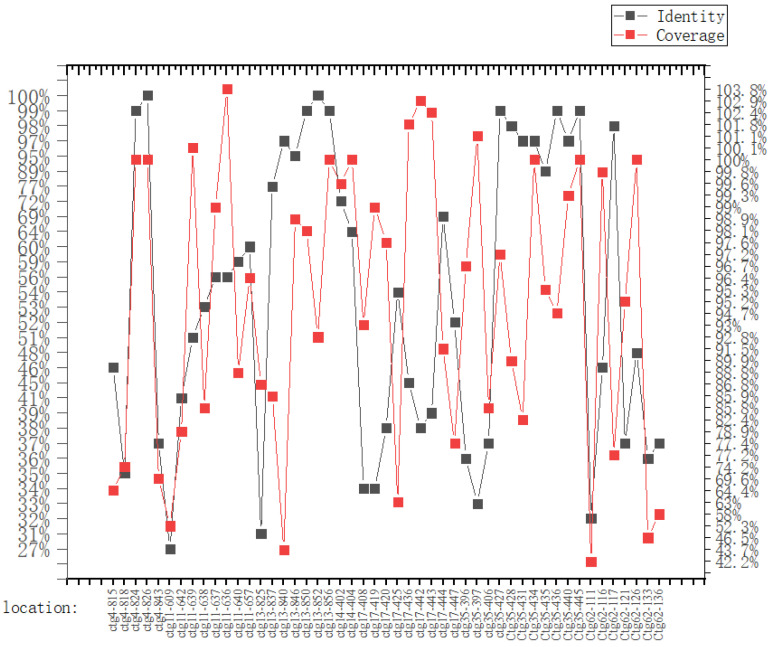
Gene location, identity and coverage of seven most similar BGCs in strain L14.

Before secondary metabolite-related BGC analysis, the whole genome sequence of strain L14 was submitted to antiSMASH database and compared with the other ten genomes of *A. niger* strains deposited in the NCBI database. The results suggested that each of these *A. niger* strains possess a similar number (at least 59) and type of BGCs (Figure 5a). Particularly, strain L14 has the largest number of BGCs classified into seven types, including PKS, NRPS, NRPS-like, terpene, indole, betalactone and siderophore (Table S3), indicating this marine strain displays a vast biosynthetic potential to produce a wide array of SMs. By sequence similarity analysis of all BGCs from these *A. niger* strains, as many as 45 SMs were predicted and summarized in the hot map (Figure 5b). All putative SMs from strain L14 were marked as blocks in red, the color depth of which positively depended on the similarity of its corresponding BGC sequence (Table S4). Further BLAST analysis resulted in characterization of seven BGCs with over 93% identity, which was, respectively, responsible for the biosynthesis of compounds TAN-1612, yanuthone D, melanin, azaniger-one A, nidulanin A and aureobasidin A and xenolozoyenone (Figure 6 and Figure 7, Tables S5–S11).

All regions 4.3, 13.2, 14.2 and 35.2 in strain L14 belong to type I PKS. Most of the functional genes in region 4.3 are similar to those in the TAN-1612 BGC from strain *A. niger* ATCC 1015 (GenBank: JN257714.1). AdaA are the core enzymes in the Ada gene cluster and had been shown to be responsible for the synthesis and cyclization of the decaketone backbone of TAN-1612 [25]. Region 13.2 displays 100% similarity with the BGC (GenBank: ACJE01000012.1), responsible for biosynthesis of yanuthone D, which is a heteroterpene derivative from polyketide 6-methylsalicylic acid (6-MSA) [26]. Region 14.2 harbors a high similar sequence with the BGC (GenBank: ACJE01000001.1) in strain ATCC 1015 for biosynthesis of melanin, which is a major ubiquitous pigment that plays an important role in photoprotection against UV radiation [27,28,29,30]. However, no hydrolase or reductase were detected in this region but phosphopantetheine-binding enzyme was. In addition, region 35.2 exhibits high similarity with the BGC (GenBank: ACJE01000001.1) responsible for biosynthesis of azanigerone A. This aza gene cluster comprises one smallest PKS domain, transacylase (SAT), C-methyltransferase (CMeT) and Zn (II)2Cys6 zinc finger transcription factor [31].

Region 11.1 is one NRPS-related BGC with a higher degree of similarity with nidulanin A BGC from *A. nidulans* FGSC A4 (GenBank: BN001308.1). This substance is synergistically synthesized by a supercluster cross, which formed with two NRPS, NlsA located on chromosome VIII and NptA located on chromosome V [32], while region 17.1 has similar ABA1 genes from *Aureobasidium pullulans* (GenBank: EU886741.1) to encode the synthetic complex of the cyclic peptide antibiotic aureobasidin A [33]. Region 62.2 has a significant BLAST hit with the gene encoding GLNRPS7 in *Glarea lozoyensis* (GenBank: KM603664.1), which is responsible for the biosynthesis of a non-ribosomal peptide xenolozoyenone. The core gene *glpks3-glnrps7* had been shown to be clustered with two transporter genes, one oxidoreductase gene and one transcriptional regulatory gene [34].

## 4. Conclusions

In this study, a high-quality whole genome of marine-derived strain L14 was obtained using next-generation and third-generation sequencing technologies. Strain L14 was undoubtedly identified as *A. niger* by morphological and *18S* rRNA sequence analyses. Genomic analysis indicated that strain L14 has one 36.1 Mb genome with a G + C content of 49.3%. Gene annotation was extensively deployed using various BLAST databases, including Nr protein sequence, Nt sequence, Swiss-Prot, GO, KEGG and COG as well as PHI and CAZy. AntiSMASH analysis uncovered 69 secondary metabolite BGCs in strain L14 and only 7 of them had high similarity with known gene clusters, suggesting most of these BGCs are unknown and yet to be unveiled. This is confirmed by our previous chemical investigation, since a great number of cryptic BGCs in strain L14 are silent or expressed at low levels under conventional conditions. These findings provide an important basis for further chemical study of strain L14 using the gene mining strategy to activate these cryptic BGCs for the production of novel bioactive SMs.

## Figures and Tables

**Table 1:** General genome features of strain L14.

Item	Value
Total length (bp)	36,104,262
Contigs	69
GC content (%)	49.3
N50 (bp)	4,203,503
N90 (bp)	2,686,881
Max-length (bp)	9,886,207

**Table 2:** Statistics of strain L14 assembly and annotation.

Type	Repeat/Copy Number	Repeat Size/Total Length (bp)	Average Length (bp)	In Genome%
DNA	82	33,600	409	0.0931
LINE	89	52,257	587	0.1447
LTR	166	79,092	476	0.2191
RC	5	2595	519	0.0072
miRNA	0	0	0	0
rRNA	80	70,794	884	0.1961
snRNA	34	4130	121	0.0114
tRNA	296	560	1	0.0016

## Data Availability

Not applicable.

## Supplementary Materials

The following supporting information can be downloaded at: https://www.mdpi.com/article/10.3390/jof8060591/s1, Figure S1: Fourteen secondary metabolites (1–14) previously isolated from strain L14; Table S1: 40 species selected to build the phylogenetic tree in NCBI database; Table S2: PHI database annotation results; Table S3: Secondary metabolite BGCs in strain L14; Table S4: BGC types of SMs of 11 *A. niger* strains. Table S5: BGC component of TAN-1612 in strain L14; Table S6: BGC component of nidulanin A in strain L14; Table S7: BGC component of yanuthone D in strain L14; Table S8: BGC component of melanin in strain L14; Table S9: BGC component of aureobasidin A in strain L14; Table S10: BGC component of azanigerone A in strain L14; Table S11: BGC component of xenolozoyenone in strain L14.
