# Supplementary material for: Genomic and AntiSMASH Analyses of Marine-Sponge-Derived Strain Aspergillus niger L14 Unveiling Its Vast Potential of Secondary Metabolites Biosynthesis
Source: J Fungi (Basel). 2022 May 31;8(6):591. doi: 10.3390/jof8060591 (PMC9224809; doi:10.3390/jof8060591)
Supplement: Supplementary file 1 [file jof-08-00591-s001.zip › jof-1718721-supplementary.pdf]

Genomic and AntiSMASH Analyses of Marine-Sponge-Derived Strain *Aspergillus niger* L14 Unveiling Its Vast Potential of Secondary Metabolites Biosynthesis

Ping Wang [1,†], Shuang Xu [1,†], Yuqi Tang [1], Hong Wang [1], Xuelian Bai [2] and Huawei Zhang [1,*]

1 School of Pharmaceutical Sciences, Zhejiang University of Technology, Hangzhou 310014, China; wangping_1009@163.com (P.W.); 17853509869@163.com (S.X.); t17670659912@163.com (Y.T.); hongw@zjut.edu.cn (H.W.)

2 College of Life and Environmental Sciences, Hangzhou Normal University, Hangzhou 311121, China; baixl2012@163.com

* Correspondence: hwzhang@zjut.edu.cn; Tel.: +86-571-88320913

† These authors contributed equally to this work.

Contents

Figure S1. Fourteen secondary metabolites (**1–14**) previously isolated from strain L14--------------------------------2

Table S1. 40 species used to build the phylogenetic tree in NCBI database--- 3

Table S2. PHI database annotation of strain L14 --4

Table S3. Secondary metabolite BGCs in strain L14 ---5

Table S4. The number and types of BGC in 11 *A. niger* strains---7

Table S5. BGC component of TAN-1612 in strain L14--8

Table S6. BGC component of nidulanin A in strain L14--9

Table S7. BGC component of yanuthone D in strain L14--10

Table S8. BGC component of melanin in strain L14--11

Table S9. BGC component of aureobasidin A in strain L14---12

Table S10. BGC component of azanigerone A in strain L14---13

Table S11. BGC component of xenolozoyenone in strain L14---14

Figure S1. Fourteen secondary metabolites (**1–14**) previously isolated from strain L14.

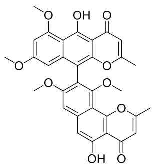
fonsecinone A (**1**)

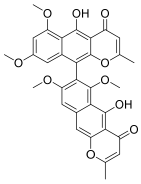
isoaurasperone A (**2**)

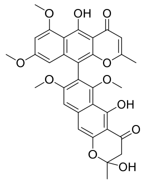
aurasperone E (**3**)

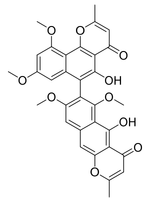
asperpyrone C (**4**)

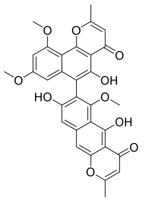
asperpyrone D (**5**)

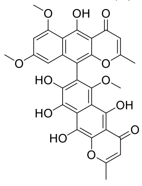
aurasperone G (**6**)

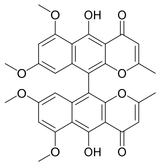
nigerone (**7**)

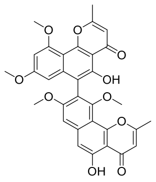
asperpyrone B (**8**)

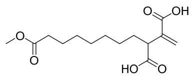
tensyuic acid E (**9**)

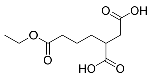
tensyuic acid F (**10**)

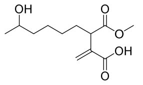
9-hydroxyhexylitaconic acid- 1-methyl ester (**11**)

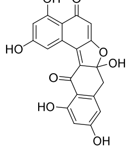
asperlone B (**12**)

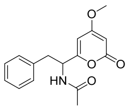
pyrophen (**13**)

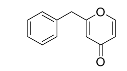
2-benzyl-γ-pyrone (**14**)

Table S1. 40 species used to build the phylogenetic tree in NCBI database.

	Strain	NCBI accession No.
1	*Aspergillus neoflavipes* CBS 260.73	NG 062800.1
2	*Aspergillus flavipes* NRRL 302	NG 063230.1
3	*Aspergillus terreus* ATCC 1012	NG 064804.1
4	*Aspergillus versicolor* NRRL 238	NG 067623.1
5	*Aspergillus nidulans* ATCC 10074	NG 064803.1
6	*Aspergillus cremeus* NRRL 5081	NG 063231.1
7	*Aspergillus proliferans* CBS 121.45	NG 064802.1
8	*Aspergillus glaucus* JCM 1575	NG 063391.1
9	*Aspergillus penicillioides* NRRL 4548	NG 063229.1
10	*Aspergillus niger* CBS 554.65	NG 065763.1
11	*Monascus pilosus* IFO 4480	NG 064816.1
12	*Xerochrysium xerophilum* CBS 153.67	NG 062798.1
13	*Xeromyces bisporus* CBS 236.71	NG 062771.1
14	*Aspergillus sclerotialis* CBS 366.77	NG 065493.1
15	*Aspergillus salinarum* DSM 27530	NG 065105.1
16	*Penicillium eremophilum* FRR 3338	NG 074879.1
17	*Penicillium limosum* CBS 339.97	NG 062729.1
18	*Penicillium hennebertii* CBS 334.68	NG 062614.1
19	*Penicillium taxi* CBS 206.57	NG 062613.1
20	*Penicillium malachiteum* CBS 647.95	NG 062770.1
21	*Penicillium paradoxum* IFO 8172	NG 064801.1
22	*Penicillium chrysogenum* CBS 306.48	NG 062803.1
23	*Talaromyces wortmannii* CBS 391.48	NG 062801.1
24	*Penicilliumcommune* CBS 343.51	NG 062634.1
25	*Evansstolkia leycettana* CBS 398.68	NG 062664.1
26	*Leiothecium ellipsoideum* CBS 607.74	NG 061073.1
27	*Aspergillus cervinus* NRRL 5025	NG 063232.1
28	*Pseudohamigera striata* IFO 6106	NG 062360.1
29	*Hamigera avellanea* CBS 295.48	NG 061105.1
30	*Thermoascus crustaceus* CBS 181.67	NG 062804.1
31	*Paecilomyces variotii* CBS 101075	NG 062372.1
32	*Paecilomyces niveus* CBS 100.11	NG 061072.1
33	*Sagenomella verticillata* CBS 414.78	NG 062610.1
34	*Ascospirella lutea* IFO 31753	NG 074878.1
35	*Talaromyces aerugineus* CBS 350.66	NG 062662.1
36	*Talaromyces viridis* CBS 114.72	NG 062609.1
37	*Talaromyces flavus var. flavus* CBS 310.38	NG 062802.1
38	*Pseudospiromastix tentaculata* CBS 184.92	NG 060987.1
39	*Chlamydosauromyces punctatus* UAMH 9990	NG 061019.1
40	*Arachniotus ruber* UAMH 3543	NG 061018.1

Table S2. PHI database annotation of strain L14.

Gene_ID	Name	Identity	Value	MolConn_ID	Protein ID	Pathogen_NCBI_species_Taxonomy_ID	Species	Phenotype_of_mutant
evm. model. tig0000000...	FZC35	26.40	3e-34	5630	G4N853	318829	*Magnaporthe-oryzae*	unaffected_pathogeni...
evm. model. tig0000397...	pabaA	62.42	0.0	178	Q9Y7F1	746128	*Aspergillus_fumigatus*	reduced_virulence
evm. model. tig0000001...	SPM1	62.86	0.0	2117	P58371	318829	*Magnaporthe_oryzae*	reduced_virulence
evm. model. tig0000005...	C3HC	31.52	6e-51	:4489	G4NGH0	318829	*Magnaporthe_oryzae*	reduced_virulence
evm. model. tig0000000...	CCN1	53.48	0.0	280	Q9HF03	5207	*Cryptococcus_neofomans*	reduced_virulence
evm. model. tig0000007...	FOS1	59.15	0.0	253	Q9C1Q7	746128	*Aspergillus_fumigatus*	reduced_virulence
evm. model. tig0000000...	GzMyb019	40.57	3e-146	1555	I1RDG6	5518	*Fusarium_graminearum*	unaffected_pathogeni...
evm. model. tig0000397...	ARG2	40.47	5e-133	743	I1RE63	5518	*Fusarium_graminearum*	reduced_virulence
evm. model. tig0000005...	MosT1	27.10	7e-44	4204	G4N740	318829	*Magnaporthe_oryzae*	unaffected_pathogeni...

Table S3. Secondary metabolite BGCs in strain L14.

Contig	Location	Most similar known cluster (%)	Type
1	1451058-1510075	bacillomycin D (20%)	NRPS
2	15900-62928	ankaflavin / monascin /rubropunctatine /monascorubrin (16%)	T1PKS
2	79358-100590	squalestain S1 (40%)	Terpene
2	1032583-1082970	EQ-4 (66%)	NRPS-like
2	1422814-1451295	-	*β*-lactone
3	547611-593175	-	NRPS-like + T1PKS
4	301772-345561	leucinostatin A / leucinostatin B (10%)	T1PKS
4	388123-432072	meridamycin (40%)	T1PKS
4	658115-702796	Tan-1612 (100%)	T1PKS
4	901838-946390	-	NRPS + T1PKS
5	113055-156099	-	NRPS-like
5	242061-283113	-	NRPS-like
7	86153-142842	-	NRPS
7	646762-693298	citreohybridonol(18%)	Terpene
8	27672-49654	-	Terpene
10	160987-182987	-	Terpene
10	524892-567666	phyllostictine A / phyllostictine B (20%)	NRPS+ T1PKS
11	491668-550990	nidulanin A (100%)	NRPS
12	465812-510362	Ucs 1025a (15%)	NRPS
13	255618-276589	-	NRPS-like
13	626214-671655	yanuthone(100%)	T1PKS
14	107275-128756	-	NRPS
14	276323-319814	melanin (100%)	T1PKS
14	443977-485041	-	NRPS-like
15	193663-235537	fumonisin (23%)	T1PKS
16	40858-86682	-	T1PKS
16	151124- 197947	-	Terpene + NRPS
16	362351-408815	-	T1PKS
17	284390-354079	AbT1 (100%)	NRPS
17	397600-451275	notoamide A (16%)	NRPS-like + T1PKS
17	560200-581477	-	Indole
18	211861-254914	-	NRPS-like
19	456536-499631	-	NRPS-like
21	70163-80837	-	Siderophore
21	92090- 135864	-	NRPS
21	336802-392807	-	NRPS-like + T1PKS
22	180836-222941	duclaxin (57%)	T1PKS
22	234582-274699	-	NRPS-like
22	305161-351727	-	NRPS
23	163589- 184806	-	Terpene
23	1998880-243698	monacolin K (22%)	NRPS
25	151976- 173052	-	Terpene
25	350148-392934	-	NRPS-like
27	154750-201208	-	T1PKS
30	64471- 109243	pladienolide B (25%)	T1PKS
30	111234- 163296	-	NRPS
32	241278-288354	-	NRPS
35	12935-69025	Aspirochlorine (9%)	NRPS
35	277551-356105	Azanigerone A (93%)	NRPS + T1PKS
36	11238-52230	-	T3PKS
37	153485-164099	-	Siderophore
37	229646-286135	-	NRPS
39	10738-72042	Ferrichrome (66%)	NRPS
40	51878-92434	Fusaric acid (13%)	NRPS-like
40	243721-303049	-	NRPS-like
42	102360-184012	phyllostictine A / phyllostictine B (20%)	NRPS + T1PKS
49	48242-99852	-	NRPS + T1PKS
52	66854-110354	-	NRPS-like
56	127302-166957	Curacin A (46%)	NRPS-like + T1PKS
62	25062-74573	-	NRPS
62	95963-147470	Xenolozoyenone (100%)	NRPS + T1PKS
67	17048-37860	-	Terpene
71	1-30698	-	NRPS-like
71	110739-120966	-	Bacteriocin
79	1-69761	-	NRPS

91	1-45428	Dihydroisoflavipucine /Isoflavipucine	NRPS-like
95	25631-46611	-	Indole
96	1-13918	-	Terpene
106	1-24683	Gulmirecin A (57%)	T1PKS

"-" indicates unknown.

Table S4. The number and types of BGC in 11 *A. niger* strains

BGC Types \ Quantity \ Strains		ATCC 1015	CBS 513.88	SH-2	ATCC 13496	An76	JSC-093350089	H915-1	L2	A1	MOD1-FUNGI2	RAF 106
PKS	T1PKS	12	12	14	14	14	12	13	13	13	16	17
	T3PKS	1	1	1	1	1	1	1	1	1	1	1
NRPS		18	18	18	18	13	17	18	18	18	22	15
NRPS-like fragment		12	11	12	12	10	12	11	11	10	16	13
Hybrid cluster	NRPS-like + PKS	4	4	4	4	4	5	4	4	4	-	4
	Terpene + PKS	-	-	-	-	-	-	-	-	-	-	1
	Terpene + NRPS	1	1	1	1	2	-	1	1	1	-	-
	Terpene + PKS+NRPS	-	-	-	-	1	-	-	-	-	-	1
Terpene		9	9	7	8	8	8	8	9	8	-	10
β-lactone containing protease inhibitor		1	1	1	1	1	1	1	1	1	-	1
Indole		2	2	2	2	2	2	2	2	2	2	2
Siderophore		2	2	2	2	2	2	2	2	2	2	1
Bacteriocin		1	1	1	1	-	-	1	1	-	-	-
Phosphonate		-	-	-	-	1	-	-	-	-	-	-

Table S5. BGC component of TAN-1612 in strain L14

Gene location	Description	MIBiG cluster	Identity	Coverage
ctg4-797	biosynthetic-additional	-	-	-
ctg4-798	biosynthetic-additional	-	-	-
ctg4-815	D-lactate dehydrogenase-like protein	BGC0000317	46%	64.4%
ctg4-818	-	BGC0001996	35%	74.2%
ctg4-824	adaA	BGC0000156	99%	100.0%
ctg4-826	metallo-β-lactamase-like thioesterase / Claisen cyclase	BGC0000156	100%	100.0%
ctg4-843	protein kinase Eg2-like protein	BGC0001057	37%	69.6%

Table S6. BGC component of nidulanin A in strain L14.

Gene location	Description	MIBiG cluster	Identity	Coverage
ctg11-609	putative SpaT	BGC00005111	27%	52.3%
ctg11-636	cyclic hydrophobic tetrapeptide	BGC0000357	56%	103.8%
ctg11-637	cyclic hydrophobic tetrapeptide	BGC0000357	56%	99.0%
ctg11-638	cyclic hydrophobic tetrapeptide	BGC0000357	53%	85.8%
ctg11-639	nonribosomal peptide synthase	BGC0001699	51%	100.1%
ctg11-640	nonribosomal peptide synthase	BGC0001699	59%	88.8%
ctg11-642	nonribosomal peptide synthase	BGC0001699	41%	78.9%
ctg11-657	ABC_multidrug_transporter	BGC0001699	60%	96.4%

Table S7. BGC component of yanuthone D in strain L14

Gene location	Description	MIBiG cluster	Identity	Coverage
ctg13-825	Yx01 protein	BGC00000433	31%	86.8%
ctg13-837	FAD / FMN-containing dehydrogenase	BGC0000170	77%	85.9%
ctg13-840	hypothetical protein	BGC0000170	97%	43.7%
ctg13-846	hypothetical protein	BGC0000170	95%	98.9%
ctg13-850	polyketide synthase	BGC0000170	99%	98.1%
ctg13-852	prenyltransferase	BGC0000170	100%	92.8%
ctg13-856	hypothetical protein	BGC0000170	99%	100.0%

Table S8. BGC component of melanin in strain L14

Gene location	Description	MIBiG cluster	Identity	Coverage
ctg14-402	Conidial yellow pigment biosynthesis PKS	BGC0000107	72%	99.6%
ctg14-404	Conidial yellow pigment biosynthesis PKS	BGC0000107	64%	100.0%

Table S9. BGC component of aureobasidin A in strain L14

Gene location	Description	MIBiG cluster	Identity	Coverage
ctg17-408	major facilitator superfamily domain, general substrate transporter	BGC0001205	34%	93.0%
ctg17-419	ABC transporter	BGC0001977	34%	99.0%
ctg17-420	adrC	BGC0001508	38%	97.6%
ctg17-425	aureobasidin A biosynthesis complex	BGC0000307	54%	63.0%
ctg17-436	cytochrome P450	BGC0000012	45%	101.8%
ctg17-442	cpaA	BGC0001563	38%	102.9%
ctg17-443	buaA	BGC0001978	39%	102.4%
ctg17-444	buaA	BGC0001978	69%	91.5%
ctg17-447	buaC	BGC0001978	52%	77.4%

Table S10. BGC component of azanigerone A in strain L14

Gene location	Description	MIBiG cluater	Identity	Coverage
ctg35-396	nonribosomal peptide synthase	BGC0001515	36%	96.7%
ctg35-397	nonribosomal peptide synthase	BGC0001515	33%	101.1%
ctg35-406	ABC transporter	BGC0001515	37%	85.8 %
ctg35-412	biosynthetic-additional	-	-	-
ctg35-427	hypothetical protein	BGC0001143	99%	97.2%
ctg35-428	hypothetical protein	BGC0001143	98%	89.9%
ctg35-431	FAD/FMN-containing dehydrogenase	BGC0001143	97%	82.4%
ctg35-434	monooxygenase	BGC0001143	97%	100%
ctg35-435	monooxygenase	BGC0001143	89%	95.3%
ctg35-436	monooxygenase	BGC0001143	99%	94.7%
ctg35-440	hypothetical protein	BGC0001143	97%	99.3%
ctg35-445	hypothetical protein	BGC0001143	99%	100.0%

Table S11. BGC component of xenolozoyenone in strain L14

Gene location	Description	MIBiG cluater	Identity	Coverage
ctg62- 111	hypothetical protein	BGC0000022	32%	42.2%
ctg62- 116	GLNRPS7	BGC0001187	46%	99.8%
ctg62- 117	hypothetical protein	BGC0001124	98%	77.2%
ctg62- 121	Exporter	BGC0000959	37%	95.2%
ctg62- 126	equisetin N-methyltransferase	BGC0001255	48%	100.0%
ctg62- 133	nonribosomal peptide synthase	BGC0001628	36%	46.5%
ctg62- 136	dehydrogenase	BGC0000162	37%	58.0%
